# Recurrent Klebsiella pneumoniae Bacteremia Due to Endotipsitis

**DOI:** 10.7759/cureus.39259

**Published:** 2023-05-20

**Authors:** Scott Everett, Andrew L Alejo, Katherine I Tatar, Joseph P Myers

**Affiliations:** 1 College of Medicine, Northeast Ohio Medical University, Rootstown, USA; 2 Department of Medicine, Summa Health, Akron, USA

**Keywords:** transjugular intrahepatic portosystemic shunt, case report, cirrhosis, bacteremia, transjugular intrahepatic portosystemic shunt (tips), klebsiella pneumoniae

## Abstract

Transjugular intrahepatic portosystemic shunt (TIPS) procedures are commonly performed in patients with cirrhosis to decrease the pressure in the portal system. An unusual complication of this procedure is endotipsitis, an infection of the shunt/stent with resultant sustained bacteremia caused by vegetation within the TIPS. The most commonly associated pathogens include staphylococci, enterococci, streptococci, and enteric Gram-negative bacilli. We present a patient who developed endotipsitis due to *Klebsiella pneumoniae* and presented with refractory *K. pneumoniae *bacteremia. Our patient had a progressively worsening clinical picture and after recognition of endotipsitis was transferred to another facility for liver transplantation and removal of the TIPS. Rapid diagnosis of endotipsitis in the setting of refractory bacteremia is essential to patient survival.

## Introduction

Transjugular intrahepatic portosystemic shunt (TIPS) procedures are minimally invasive and are used in patients with cirrhosis to decompress the pressure in the portal system. Complications include capsular perforation, intraperitoneal hemorrhage, stent misplacement, and endotipsitis [1]. Endotipsitis, a term first introduced by Sanyal et al. [2], is defined by two definitions: a “definite” infection occurs with continuous clinically significant bacteremia with vegetations/thrombi inside TIPS or a “probable” infection implies sustained bacteremia with no other identifiable source of infection in a patient with TIPS [3]. Because of these relatively vague definitions, it is difficult to know the exact incidence of the disease. A single-center retrospective study done in 2010 revealed a 1% incidence at that specific institution [3]. The most common etiologic agents for endotipsitis are enteric Gram-negative bacilli, enterococci, staphylococci, and streptococci [4].

*Klebsiella pneumoniae* is an enteric Gram-negative bacillus commonly associated with intraperitoneal infections and pneumonia in patient populations with alcohol use disorder and diabetes [5]. The bacterium colonizes human mucosal surfaces of the oropharynx and gastrointestinal tract and infects patients requiring ventilators or intravenous catheters [6]. In a 2009 review of endotipsitis, *K. pneumoniae* accounted for two of 40 (5%) reported cases [1]. *K. pneumoniae* has recently developed resistance genes to code for multi-drug resistance, including third-generation cephalosporins and carbapenems [7]. In the healthcare setting, *Klebsiella* is spread via contamination of the hands of healthcare personnel or other individuals closely associated with an infected patient [8].

In this report, we present a case of endotipsitis diagnosed in the setting of recurrent *K. pneumoniae* bacteremia in a patient with decompensated alcoholic cirrhosis, portal hypertension, and the presence of a TIPS.

## Case presentation

A 38-year-old Caucasian male patient with acute decompensated alcoholic liver cirrhosis presented to the emergency department (ED) with complaints of hematemesis. This patient was status post TIPS procedure two years previously with a model for end-stage liver disease-sodium (MELD-Na) score of 35. This score is indicative of a 65% 90-day mortality rate. He denied alcohol use in the last eight months and was on the liver transplant list at multiple facilities. Because of the hematemesis, he was admitted and underwent endoscopic evaluation. He was found to have esophageal variceal hemorrhage and was treated with intravenous octreotide acetate. He was not treated with variceal banding due to his esophageal anatomy displaying extensive scarring and focal evagination of the lower esophagus. He was also given pantoprazole and intravenous ceftriaxone for the prevention of spontaneous bacterial peritonitis (SBP). *K. pneumoniae* was found on BioFire FilmArray (BioFire Diagnostics, Salt Lake City, Utah) analysis of both admission blood cultures and was isolated in culture from both initial blood cultures specimens. Once susceptibility testing was available, ceftriaxone was changed to cefazolin to complete eight days of parenteral therapy followed by five days of oral cephalexin.

**Figure 1:**
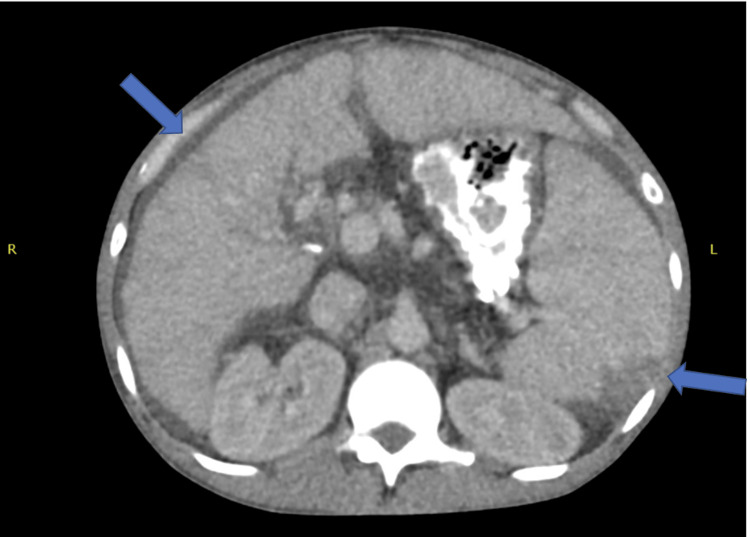
Computed tomography revealing a wedge-shaped hypodensity in the spleen representing infarction, moderately large amount of ascites surrounding the liver, and irregularity of the liver contour demonstrating cirrhosis of the liver.

**Figure 2:**
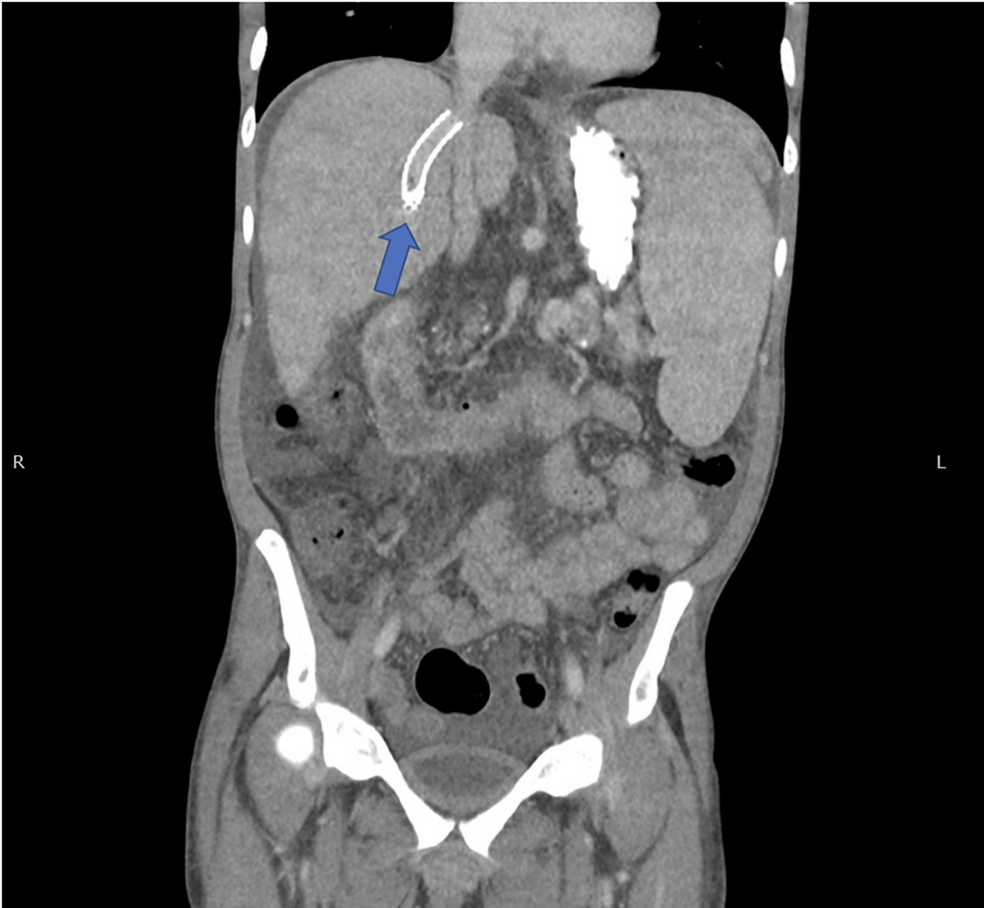
Computed tomography imaging demonstrating patency of the transjugular intrahepatic portosystemic shunt catheter.

The patient initially improved and was transferred from the intensive care unit to the medical floor. After completion of antimicrobial therapy, the patient again deteriorated clinically with leukocytosis, fever, abdominal distention, and mild diarrhea. Workup for *Clostridium difficile* disease was negative but repeat blood cultures were again positive for *K. pneumoniae*. Piperacillin-tazobactam was begun and computed tomography (CT) of the chest, abdomen, and pelvis revealed ground glass opacities of the lung, hepatic cirrhosis, abdominal esophageal varices, a large number of ascites, and an area of splenic infarction (Figure 1). An ultrasound was then obtained to demonstrate the patency of the TIPS catheter, with the appropriately directed flow in all vessels excluding the left portal vein, which demonstrated some retrograde flow that can be expected post-TIPS procedure, with no vegetations in the stent shown in the CT (Figure 2). Transthoracic echocardiogram was negative for evidence of endocarditis. In the absence of any other site for recurrent bacteremia, he was diagnosed with probable endotipsitis. While being treated with a continued course of parenteral piperacillin-tazobactam, he was accepted for liver transplantation at another facility and was transferred there in improved condition.

Treatment and outcomes

Intravenous ceftriaxone was initially administered to prevent SBP. When *K. pneumoniae* bacteremia was documented and susceptibilities were available, antimicrobial therapy was de-escalated to intravenous cefazolin and then eventually to oral cephalexin. Due to the progressive nature of the *Klebsiella* bacteremia, the patient began to develop worsening hepatic encephalopathy and therefore was begun on lactulose and rifaximin per standard protocol. Diagnostic aspiration of peritoneal fluid was non-diagnostic. After recurrent bacteremia due to probable endotipsitis was documented, piperacillin-tazobactam was administered but the patient continued to clinically decline with worsening hepatic encephalopathy. This prompted a transfer to a facility with liver transplantation capabilities.

## Discussion

This case emphasizes the diagnosis of endotipsitis as an emerging prosthesis-associated infection in patients with TIPS. Given the severity of this disease and the significant mortality, endotipsitis should be considered in any patient post-TIPS with recurrent bacteremia not explained by other common causes (endocarditis, indwelling catheters, pneumonia). Endotipsitis may also occur as a result of a fungal infection. *Candida*, a fungal pathogen described in other endotipsitis cases, has been associated with the highest mortality (>60%) [9]. Prompt antibiotic or antifungal treatment is warranted to help decrease additional complications and mortality. Early endotipsitis occurs within 120 days of placement, while late endotipsitis occurs after 120 days of placement. Early endotipsitis usually occurs as a result of bacterial seeding during the initial procedure contrasted with late endotipsitis most commonly occurring after a TIPS revision surgery. Until 2017, only 56 cases have been reported with a mortality of 32% [10].

Our patient developed* K. pneumoniae* bacteremia without an identifiable primary source that relapsed after discontinuation of the initial course of antimicrobial therapy. No other primary source for the *K. pneumoniae* bacteremia was found, so the diagnosis of “probable endotipsitis” was made. A diagnosis of “definite endotipsitis” would have required vegetations to have been seen on either a CT scan or ultrasound of the TIPS. The development of hepatic encephalopathy with the emergence of recurrent *K. pneumoniae* bacteremia prompted a transfer to a liver transplant-capable facility. TIPS devices can only be removed surgically and would have been removed at the time of liver transplantation.

The diagnosis of endotipsitis is a diagnosis of exclusion but must be considered in any patient who has a TIPS and who has recurrent or refractory bacteremia in the face of appropriate antimicrobial therapy. Because it is difficult to visualize TIPS-related vegetations on CT or ultrasound, endotipsitis may be underdiagnosed. A study done by Bouza et al. showed that the average time for endotipsitis to occur was 210 days, with a range of six to 732 days [11]. Endotipsitis usually presents primarily with fever, which may be accompanied by malaise, chills, anorexia, and right upper quadrant pain [12]. Some case reports have also described liver dysfunction causing variceal bleeding similar to that seen in our patient. These authors also treated patients with presumptive or proven endotipsitis with empirical antibiotics and suggested that eligible patients should be referred for liver transplantation, as in our patient. Perioperative prophylactic antibiotics during the initial TIPS procedure are controversial and of unproven efficacy. However, as with joint, valve, and other endoprostheses, perioperative antimicrobial prophylaxis is common practice [1].

## Conclusions

We report a patient with endotipsitis due to *K. pneumoniae* that was only suspected after the patient developed recurrent *K. pneumoniae *bacteremia in the setting of appropriate therapy for the first episode of* K. pneumoniae* bloodstream infection. Our patient, with a known TIPS procedure two years previously, presented with hematemesis, which led to an eventual diagnosis of bacteremia. CT of the chest, abdomen, and pelvis along with blood cultures were able to successfully identify the pathogen involved, as well as help guide antimicrobial therapy. Endotipsitis should be suspected in any patient who has a TIPS and who develops bacteremia without an identifiable source.
